# Two stable gut microbiome guilds predict liver tumor class and treatment responses

**DOI:** 10.1002/imt2.70123

**Published:** 2026-04-02

**Authors:** Yang Liu, Zefan Zhang, Guojun Wu, Bowen Li, Linghua Wang, Jincheng Wang, Zixian Wei, Zhiyue Wang, Jinhua Yang, Kunyu Zhang, Tianqi Zhang, Xin Tao, Tao Chen, Jia Fan, Jian Zhou, Xinrong Yang, Liping Zhao, Yunwei Wei

**Affiliations:** ^1^ Department of Hepatobiliary and Pancreatic Surgery Division, Ningbo No. 2 Hospital Wenzhou Medical University Ningbo China; ^2^ Ningbo Key Laboratory of Intestinal Microecology and Human Major Diseases Ningbo China; ^3^ Department of Hepatobiliary Surgery & Transplantation, Liver Cancer Institute, Zhongshan Hospital Fudan University; Key Laboratory of Carcinogenesis and Cancer Invasion, Ministry of Education Shanghai China; ^4^ Department of Biochemistry and Microbiology, School of Environmental and Biological Sciences and Center for Microbiome, Nutrition, and Health, New Jersey Institute for Food, Nutrition, and Health, Rutgers The State University of New Jersey New Brunswick New Jersey USA; ^5^ Rutgers‐Jiaotong Joint Laboratory for Microbiome and Human Health New Brunswick New Jersey USA; ^6^ State Key Laboratory of Microbial Metabolism and Ministry of Education Key Laboratory of Systems Biomedicine, School of Life Sciences and Biotechnology Shanghai Jiao Tong University Shanghai China

**Keywords:** hepatocellular carcinoma, gut microbiome, microbiome signatures, two competing guilds

## Abstract

Gut microbiome alterations are increasingly associated with hepatocellular carcinoma (HCC), highlighting the gut–liver axis as a key contributor to tumor progression and prognosis. Taxon‐based HCC microbiome studies have shown limited reproducibility because they are affected by database dependency, taxonomic ambiguity, and overlooked ecological interactions. The Two Competing Guilds (TCG) model, based on stable gut microbiome interactions, provides a structurally grounded framework for robust, generalizable biomarkers. Using shotgun metagenomic data from a newly recruited cohort of 120 surgically resectable HCC cases and 76 benign liver tumor controls, we constructed co‐abundance networks to identify stably correlated genome pairs and assembled a hepatic cancer‐TCG (HCC‐TCG) model composed of 142 genomes. Functionally, one Guild had more genes for butyrate production from carbohydrate fermentation while the other Guild was enriched in genes for virulence factors and antibiotic resistance, highlighting its potential proinflammatory roles. Classifiers trained on the abundance profiles of HCC‐TCG genomes successfully distinguished HCC from benign liver tumors (area under the receiver operating characteristic, AUROC = 0.70) and from colorectal liver metastases (CRLM) (AUROC = 0.78). In an external validation cohort, the model further discriminated against HCC from intrahepatic cholangiocarcinoma (iCCA) (AUROC  =  0.72), and from healthy controls (AUROC  =  0.79–0.85), demonstrating its broad applicability for tumor stratification across clinical contexts. Moreover, HCC‐TCG profiles predicted post‐resection recurrence risk and response to adjuvant therapies (AUROC up to 0.83). Importantly, external validation in two independent cohorts of advanced HCC patients treated with PD‐1/PD‐L1 inhibitors demonstrated consistent predictive performance (AUROC  =  0.64–0.73), confirming the model's generalizability in nonsurgical and immunotherapy contexts. This genome‐specific, ecologically structured, and database‐independent framework identifies a conserved Guild‐based microbiome signature for HCC. Our findings demonstrate that a fixed genome‐resolved ecological structure retains transferable discriminatory signal across clinical contexts. The HCC‐TCG framework provides a genome‐specific, interaction‐based foundation for future development of non‐invasive microbiome stratification strategies requiring prospective validation.

## Highlights

- A genome‐resolved, database‐independent framework defines the core microbiome as two competing guilds with stable ecological connectivity in hepatocellular carcinoma (HCC) patients.
- The HCC‐two competing guilds (HCC‐TCG) signature enables robust liver tumor stratification and predicts recurrence and immunotherapy response across independent cohorts.
- Guild‐based modeling overcomes taxonomic ambiguity, delivering a reproducible and clinically actionable signature for precision oncology in HCC.

## Graphical abstract

Gut microbiome alterations are linked to hepatocellular carcinoma (HCC), yet taxon‐based biomarkers often lack reproducibility. Using shotgun metagenomics from 120 resectable HCC (MT) and 76 benign hepatic tumor controls (BT), we reconstructed a stable interaction‐based Two Competing Guilds model (HCC‐TCG) comprising 142 genomes organized into two antagonistic guilds. The ecological guild balance distinguished HCC from benign and other hepatic tumor types, generalized across external cohorts, and predicted post‐resection recurrence and adjuvant therapy response. Validation in advanced HCC immunotherapy cohorts further supported its transferability, establishing a genome‐resolved ecological signature for tumor stratification and noninvasive risk prediction.

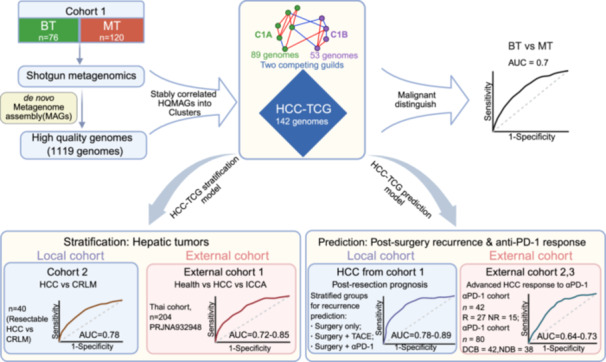

## INTRODUCTION

Hepatocellular carcinoma (HCC) is a leading cause of cancer‐related death worldwide, and China bears the heaviest global burden, accounting for nearly 50% of all new cases [1, 2]. Most patients are diagnosed at an advanced stage, and many experience early recurrence even after curative‐intent surgery, resulting in a 5‐year overall survival rate of less than 14.4% in China [3]. Even among patients who undergo potentially curative resection, postoperative recurrence remains a major unmet clinical challenge, with recurrence or metastasis occurring in approximately 60%–70% of patients within 5 years [4]. Despite advances in surgical techniques, locoregional therapies, and the introduction of immune checkpoint inhibitors (ICIs), overall clinical outcomes for HCC remain unsatisfactory [4, 5].

HCC patients with similar baseline clinical parameters and tumor stage often have markedly different prognoses: some remain disease‐free for years, whereas others develop recurrence within months or fail to respond to immunotherapy [6, 7]. These unexplained clinical divergences suggest the existence of additional, yet‐unidentified biological factors—beyond tumor genomics and conventional clinical characteristics—that shape the evolution of HCC and its responsiveness to therapy. Increasing studies indicate that the gut microbiome, connected to the liver through the portal circulation, is a major ecological driver of hepatic inflammation, immune responses, and oncogenesis [8].

Increasing evidence indicates that gut microbial dysbiosis is closely involved in hepatocarcinogenesis and that specific microbial signatures can serve as non‐invasive biomarkers for early HCC detection and for predicting response to anti‐PD‐1 immunotherapy [9, 10, 11]. Despite growing interest in the gut–liver axis, clinically annotated HCC gut metagenomic cohorts remain limited, and most publicly available datasets are dominated by late‐stage, surgically unresectable disease [12, 13]. Microbiome signatures with direct relevance to surgical pathways, including pretreatment stool sampling, postoperative adjuvant therapy documentation, and longitudinal outcomes are still scarce. Here, we address this gap in a resectable, surgery‐based HCC cohort with structured follow‐up. In addition, as has been observed in gut microbiome studies across multiple disease contexts, HCC‐related microbiome research remains fragmented, and substantial inconsistencies across studies highlight a core bottleneck that has hindered the meaningful translation of these findings into clinical practice [14]. The widely used taxon‐based analyses often identify “HCC‐associated” genera or species in individual cohorts, yet such signatures derived from one population rarely generalize to others. These issues stem from several inherent limitations. First, by relying on predefined reference databases, they inevitably exclude novel or unclassifiable microbial entities that often contain valuable biological information [15]. Moreover, they collapse strain‐level genomic and functional diversity into coarse categories such as “genus” or “species,” obscuring differences in metabolic pathways, virulence, or ecological behavior [16, 17]. In addition, by being typically identified as independent units, bacterial ecological interactions are always ignored—such as cooperation and competition, which underpin community ecological characteristics. These limitations make it difficult to extract reproducible or mechanistically interpretable microbial patterns relevant to HCC.

To overcome these challenges, we developed a genome‐resolved, Guild‐based analytical framework that focuses on microbial genomes and their ecological relationships rather than on disease‐associated taxa [18]. By reconstructing high‐quality metagenome‐assembled genomes (HQMAGs) de novo, we capture all prevalent microbial genomes—including novel and unclassified ones—with strain‐level resolution. By retaining genome content, we preserve functional heterogeneity that is lost when collapsing organisms into taxonomic ranks. And by analyzing stable correlations among genomes instead of their isolated abundances, we identify groups of microbes as Guilds that act together consistently across biological perturbations, providing a robust ecological basis for discovering clinically relevant microbiome structures.

Using this approach, our recent study identified a set of stably connected genomes that consistently co‐varied across dietary interventions and 15 distinct human diseases [18]. These genomes are self‐organized into two competing guilds (TCGs) together forming a core structural backbone of the human gut microbiome. Notably, the abundance of these core genomes alone was sufficient to train machine‐learning models that accurately classified cases from controls across 15 distinct human diseases and predicted patient responses to immunotherapy across four diseases including two cancer cohorts. These findings established that a stability‐defined Guild structure captures clinically meaningful microbial signals that taxon‐based methods fail to detect and suggested that similar structures might exist in specific diseases, including HCC.

Here, we asked whether the shift from benign to malignant liver tumors could be used as a new ecological perturbation to reveal a stable Guild structure specific to HCC. Specifically, we examined if prevalent gut microbial genomes maintain stable relationships across benign and malignant states, and whether these stably connected genomes self‐organize into a TCG architecture—a hepatic tumor‐specific “HCC‐TCG”. We then evaluated whether this HCC‐TCG structure distinguishes benign from malignant tumors (MTs), generalizes across primary and metastatic liver malignancies, and predicts early recurrence after surgery, response to anti‐PD‐1 or transarterial chemoembolization (TACE) therapy in independent cohorts.

In this study, we reconstructed 1119 high‐quality genomes from preoperative fecal samples and identified 142 genomes whose interactions remained stable across benign and malignant liver tumors. These genomes formed two functionally contrasting Guilds—together constituting the HCC‐TCG. We show that this stable Guild structure distinguishes multiple forms of liver malignancy, predicts early postoperative recurrence, and forecasts response to anti‐PD‐1 or TACE adjuvant therapy following surgery in advanced HCC cohorts. By shifting the analytic focus from fluctuating taxa to stable genome‐level relationships, this work provides a reproducible, mechanistically interpretable microbial signature of liver cancer and establishes the foundation for Guild‐based risk assessment and therapeutic strategies in HCC.

## RESULTS

### Two stably competing microbiome guilds identified in patients with liver tumors

**Figure 1:**
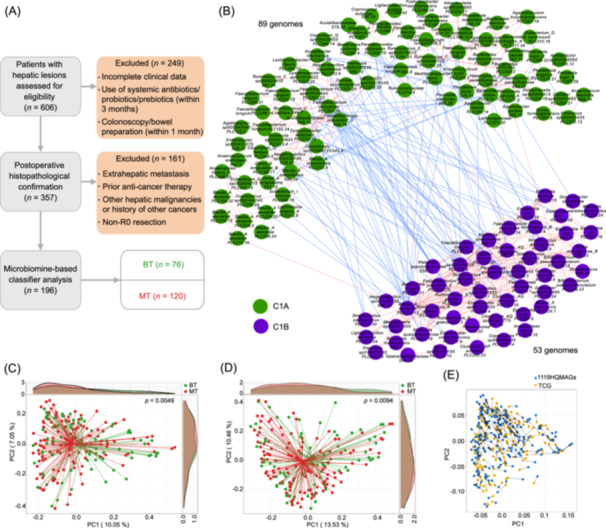
Overall variations in the gut microbial composition and the two competing guilds (TCG) associated with hepatocarcinogenesis. (A) Study flow chart and sample inclusion for microbiome‐based classifier analysis. Patients with hepatic lesions assessed at Zhongshan Hospital were screened, with exclusions due to incomplete clinical data, recent systemic antibiotics/probiotics/prebiotics (within 3 months), or recent colonoscopy/bowel preparation (within 1 month), as well as hepatocellular carcinoma (HCC)‐specific exclusions (extrahepatic metastasis, prior anticancer therapy, other hepatic malignancies/history of other cancers, or non‐R0 resection). The final classifier dataset included benign tumors (BT, *n* = 76) and malignant tumors (MT/HCC, *n* = 120). (B) The stable network of the TCG, C1A 89 high‐quality metagenome‐assembled genomes (HQMAGs) and C1B 53 HQMAGs, the red lines indicate positive correlations, while the blue lines represent negative correlations. (C) Principal Coordinate analysis (PCoA) based on Bray–Curtis distance calculated from 1119 genomes. (D) PCoA analysis based on Bray–Curtis distance calculated from 142 TCG genomes. (E) Procrustes analysis was performed on the PC scores derived from PCoA ordination based on Bray–Curtis dissimilarities calculated from 1119 genomes and 142 TCG genomes. Procrustes sum of squares = 0.09, *p* = 0.001.

To explore gut microbiome differences associated with malignant transformation, we metagenomically sequenced fecal samples collected after admission but preoperatively from 120 patients with pathologically confirmed HCC who later underwent surgical resection (MT group) and 76 patients diagnosed with benign liver tumors, such as hepatic hemangioma, based on postoperative pathology (benign tumor, BT group) (Table S1). As shown in the cohort flow diagram (Figure 1A), all patients were prospectively enrolled during their hospitalization, underwent standardized preoperative evaluation, and provided fecal samples under uniform protocols. This ensured clinical comparability between groups while minimizing sampling bias.

We de novo reconstructed 1119 nonredundant HQMAGs, merging any two HQMAGs into one if their ANI was over 99%. These HQMAGs represented over 74.43% of the total sequenced metagenomic reads and served as the primary microbiome features for further analysis. Alpha‐diversity of the gut microbiota, measured by Shannon Index, was similar between the MT and BT groups (Figure S1, *p* = 0.47). Regarding beta‐diversity, principal coordinate analysis (PCoA) and PERMANOVA test based on Bray–Curtis distance revealed a significant difference in microbial composition between the two group (*p* = 0.0049), indicating a distinct gut microbiome associated with hepatocarcinogenesis (Figure 1C).

We next sought to identify stably correlated microbes from prevalent HQMAGs that could form the basis of a Guild‐level ecological model. A total of 206 HQMAGs, which were shared by more than 75% of the samples in both the MT and BT groups were selected. Out of the 21,115 possible genome pairs, a vast majority (86.96%) showed no significant correlation in either group, consistent with the idea that ecological interactions may constitute a small subset of the core gut microbiome [19]. However, 372 genome pairs exhibited stable correlations—defined as preserving both direction and statistical significance across the MT and BT groups—suggesting persistent ecological interactions (Figure S2).

To explore how these stable interactions were organized at the community level, we next examined their network structure. Connected components clustering analysis organized these stably correlated HQMAGs into 2 distinct unconnected clusters (Figure S3). Cluster C1 included 142 HQMAGs with both positive and negative stable correlations, whereas Cluster C2 was composed of only two positively correlated HQMAGs. Weighted correlation network analysis (WGCNA) based on average linkage tree built upon stable correlations further divided C1 into two sub‐clusters, C1A with 89 and C1B with 53 HQMAGs, respectively. C1A and C1B displayed internally cooperative but mutually antagonistic relationships as evidenced by 84.46% and 99.26% stable correlations within C1A and C1B were positive, while 94.25% inter‐cluster correlations were negative (Figure 1B). This pattern was consistent with the (TCG) structure found in our previous work [18]. Although Guild definition is connectivity‐based rather than taxonomy‐driven, the 142 HQMAGs span 5 phyla, with Bacillota dominant in both guilds (76.40% in C1A and 64.15% in C1B). C1A and C1B had remarkable differences in Actinomycetota, Bacteroidota, and Pseudomonadota (Table S2). This demonstrates that guild structure reflects cross‐taxonomic ecological organization rather than taxonomic aggregation.

Hereafter, we referred C1A and C1B collectively as HCC‐TCG in this study. Beta diversity analysis based on the 142 HCC‐TCG HQMAGs revealed significant divergence between the MT and BT groups (*p* = 0.0094) (Figure 1D). Notably, variation within the 142 HCC‐TCG genomes recapitulated the principal beta diversity pattern observed when using the full set of 1119 HQMAGs. To further assess this, we performed Procrustes analysis, a technique for quantifying concordance between two ordination results in multidimensional space. The analysis revealed a strong concordance between the two datasets (Procrustes sum of squares = 0.09, *p* = 0.001), indicating that the HCC‐TCG subset retains the dominant ecological structure of the full microbial community. This was further supported by a Mantel test (*R* = 0.88, *p* = 0.001), confirming that the HCC‐TCG signature captures the major variation in gut microbial composition across samples (Figure 1E).

### Distinct genetic functional profiles of the two guilds in HCC‐TCG

**Figure 2:**
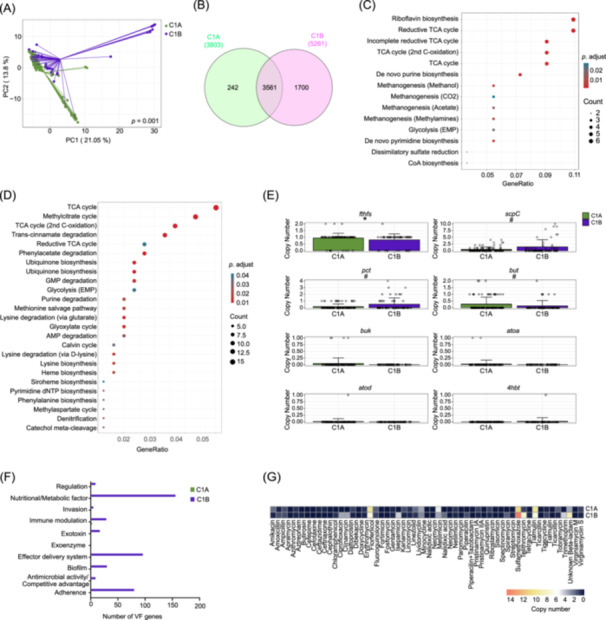
Distinct functional genetic profiles between C1A and C1B. (A) PCoA plot based on the Euclidean distance among KOs (KEGG Orthologs) showed significant separations between C1A and C1B of the HCC‐TCG. (B) The Venn diagram showed the number of shared and unique KOs in C1A and C1B. (C) Enrichment analysis of KOs with significantly higher copies in C1A by KEGG modules. (D) Enrichment analysis of KOs with significantly higher copies in C1B by KEGG modules. (E) Bar plot showing the copy numbers of genes encoding *fthfs* (formate–tetrahydrofolate ligase) for acetate production; *scpC* (propionyl‐CoA: succinate‐CoA transferase) and *pct* (propionate‐CoA transferase) for propionate production; and *but* (butyryl‐CoA: acetate CoA transferase), *buk* (butyrate kinase), *4hbt* (butyryl‐CoA: 4‐hydroxybutyrate CoA transferase), and *ato* (butyryl‐CoA: acetoacetate CoA transferase; *atoa*, alpha subunit; *atod*, beta subunit) for butyrate production. Differences between C1A and C1B were assessed using the two‐sided Mann–Whitney *U* test. (F) Bar plot showed the number of virulence factors (VF) genes and the corresponding VF classes. (G) Heatmap plot showed the copy numbers of drug resistance gene to different antibiotics. Each column represents one antibiotic, colors indicate gene copy number, ranging from dark blue (0 copies) to red (14 copies), as shown in the color bar. ^#^ *p* < 0.1, *p < 0.05, **p < 0.01, ***p < 0.001.

To elucidate the functional differences between the two Guilds identified in the HCC‐TCG, we first performed non‐targeted functional annotation on the 142 HQMAGs from both C1A and C1B based on KEGG ortholog (KO). In total, 3803 and 5261 KOs were identified in C1A and C1B, respectively. Significant KOs between the two guilds were identified by Mann–Whitney test, *p* values were adjusted by Benjamini–Hochberg method. PCoA analysis and PERMANOVA test based on Euclidean distance calculated from the total KO profiles showed distinct genetic functions between the two Guilds (Figure 2A, *p* = 0.001). Among all the KOs, 242 and 1700 were unique in C1A and C1B, respectively (Figure 2B). In addition, 2239 KOs had significant differences in copy numbers between the two Guilds, 231 were higher in C1A and 2008 were higher in C1B. Enrichment analysis showed that the KOs with higher copies in C1A were enriched in 14 modules, including 4 related to methane production (Figure 2C). Those with higher copies in C1B were enriched in 25 modules, related to energy metabolism (e.g., TCA cycle, glyoxylate cycle), nucleotide turnover, aromatic compound, and short‐chain fatty acid (SCFA) precursor metabolism (Figure 2D).

We then conducted a targeted comparison of several human health‐related microbial functional characteristics between C1A and C1B. SCFAs are reported to have anti‐inflammatory and anti‐cancer properties. We examined the distribution of terminal genes involved in butyrate biosynthesis across the two Guilds. Among these, C1B exhibited significantly higher copy numbers of *pct* and *scpC*, suggesting an enhanced potential for butyrate synthesis. In contrast, the *fthfs* and but genes tended to be more abundant in C1A, whereas *buk*, *atoA*, *atoD*, and *4hbt* did not differ significantly between the two Guilds (Figure 2E). These findings reflect a Guild‐specific divergence in butyrate biosynthetic capacity, with C1B enriched in alternate pathways potentially linked to proteolytic fermentation.

To compare the functional basis related to the potential detrimental effect of gut microbiota on human health, we identified virulence factor genes (VFs) and antibiotic resistance genes (ARGs) in the genomes of HCC‐TCG. C1B had 423 VFs genes from 10 VF classes and 83 ARGs related to 48 antibiotics while C1A had 3 VFs genes involved in 2 VF classes and 63 ARGs related to 46 antibiotics (Figure 2F,G). C1B showed higher genetic capacity to adapt to antibiotic exposure and greater potential to disrupt host immune surveillance. By contrast, C1A exhibited minimal representation in virulence categories, consistent with a less invasive, potentially homeostatic ecological role. In conclusion, these results indicated that the two Guilds in the HCC‐TCG have distinct genetic capacities, with C1A potentially protective and C1B detrimental.

### HCC‐TCG as a microbiome signature to classify hepatic tumors

**Figure 3:**
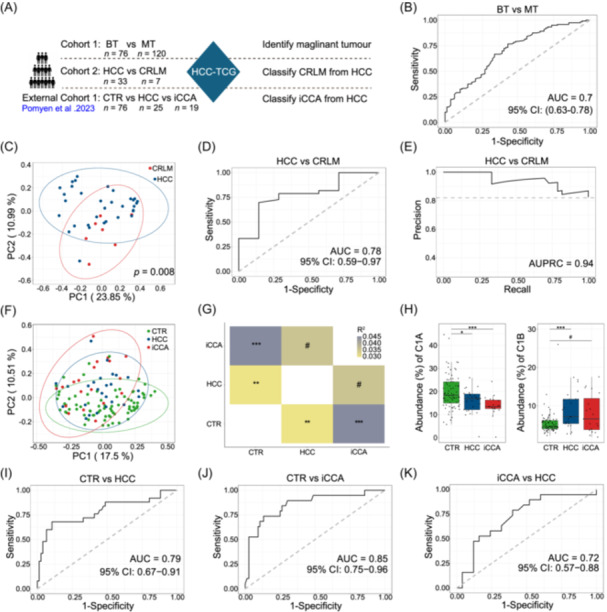
HCC‐TCG as a signature to classify patients with different hepatic tumors. (A) Overview of study design showing three datasets used for classification: two internal cohorts (Cohort 1: benign tumor, BT vs. malignant tumor, MT; Cohort 2: HCC *n* = 33 vs. colorectal liver metastases, CRLM *n* = 7) and one external cohort (health control, CTR *n* = 76, HCC *n* = 25, intrahepatic cholangiocarcinoma, iCCA *n* = 19). (B) Based on the abundance matrix of the 142 HQMAGs of HCC‐TCG, a Random Forest classification model with leave‐one‐out cross‐validation was trained to classify MT and BT patients. The area under the receiver operating characteristic (AUROC) is shown. (C) PCoA plot based on Bray‐Curtis distances calculated from the 142 HQMAGs of HCC‐TCG, showing separation between HCC and CRLM groups. (D) Receiver operating characteristic (ROC) curve for a Random Forest classification model distinguishing between CRLM and HCC with AUROC. (E) Precision‐Recall curve for the same model, with area under the precision–recall curve (AUPRC). (F) PCoA plot between groups based on the Bray‐Curtis calculated from the 142 HQMAGs of HCC‐TCG. (G) Pairwise PERMANOVA test between the HCC, iCCA, and health control groups. (H) Boxplot of the abundance of C1A and C1B between the HCC, iCCA, and CTR groups. Boxes show medians and interquartile ranges (IQRs); whiskers denote the lowest and highest values that were within 1.5× the IQR from the first and third quartiles, and outliers are shown as individual points. Kruskal‐Wallis test followed by Dunn's post hoc test (two‐sided) was used to compare groups. (I–K) Based on the abundance matrix of the 142 HQMAGs of HCC‐TCG, Random Forest classification models with leave‐one‐out cross‐validation were trained to classify CTR versus HCC, CTR versus iCCA, iCCA versus HCC. The Classifier performance was assessed by AUROC. ^#^ *p* < 0.1, *p < 0.05, **p < 0.01, ***p < 0.001.

To assess the classification potential of the HCC‐TCG on different liver tumor types, we first applied the abundance of the 142 HQAMGs in C1A and C1B as input features to train a Random Forest classifier for distinguishing malignant hepatic tumor (Figure 3A). The resulting model demonstrated a moderate performance to distinguish MT patients from BT subjects, with an AUROC of 0.70 (Accuracy = 0.68) (Figure 3B), suggesting that the HCC‐TCG captures core gut microbiome characteristics shifts associated with malignant transformation. To further evaluate the generalizability of the HCC‐TCG model across different hepatic tumor types, we next analyzed another internal clinical cohort (Cohort 2, Figure 3A) consisting of HCC (*n* = 33) patients and CRLM (*n* = 7) patients, representing primary and secondary hepatic malignancies (Table S3). All 142 HQMAGs of HCC‐TCG had an estimated abundance in this dataset, with an average prevalence of 80.60%. These HQMAGs accounted for 26.16% of the total abundance in this dataset. PCoA based on Bray–Curtis distances revealed distinct clustering between HCC and CRLM (PERMANOVA, *p* = 0.008) (Figure 3C). Using the abundance profiles of the 142 HQMAGs as input features, a Random Forest classifier achieved an AUROC of 0.78 (Accuracy = 0.825) (Figure 3D) and an AUPRC of 0.94 (Figure 3E) for distinguishing HCC from CRLM, demonstrating good discriminative power. However, given the limited sample size in the CRLM, analyses involving this group should be interpreted with caution.

In addition to these two internal cohorts, we further estimated the abundance of the 142 HQMAGs defining the HCC‐TCG in an independent external shotgun metagenomic dataset from Thailand population that included HCC patients (*n* = 25), iCCA (*n* = 19), and healthy controls (CTR, *n* = 76) (Figure 3F) [20]. All 142 HQMAGs of HCC‐TCG had an estimated abundance in this dataset, with an average prevalence of 90.85%. These HQMAGs accounted for 24.63% of the total abundance. PCoA revealed distinguishable separation CTR, HCC, and iCCA (Figure 3G). Consistently, pairwise PERMANOVA based on Bray–Curtis distances confirmed significant compositional differences between the group (Figure 3H). The summed abundance of HQMAGs in both C1A and C1B showed no difference between disease groups, that is, HCC and iCCA, which is similar to what we found between MT and BT (Figure S4), as well as HCC and CRLM (Figure S5). However, the C1B Guild—previously associated with virulence, antibiotic resistance, and metabolic flexibility—was significantly enriched in both HCC and iCCA patients compared to controls. In contrast, the C1A Guild was less in tumor‐bearing individuals, supporting its role as a potentially protective or homeostatic microbial group (Figure 3I). Random Forest classifiers built on HCC‐TCG effectively distinguished HCC and iCCA from healthy controls with AUROC values of 0.79 (Accuracy = 0.86) and 0.85 (Accuracy = 0.84) respectively (Figure 3J). Moreover, although C1B is elevated in both HCC and iCCA (with iCCA showing a stronger shift toward C1B dominance), HCC‐TCG showed a moderate ability to discriminate iCCA from HCC (AUROC = 0.72, Accuracy = 0.70), offering potential clinical value in a setting where preoperative differentiation between these two entities is often challenging using conventional clinical and imaging assessments (Figure 3K). Notably, this discrimination is not driven by C1B alone; rather, the classifier leverages genome‐resolved multivariate patterns across the 142 HQMAGs, including the C1A–C1B balance and within‐Guild genome abundance configurations, which together enable separation of iCCA from HCC despite a shared malignant‐associated Guild shift. These findings indicate that HCC‐TCG captures tumor‐associated microbiome shifts and supports its use as a genome‐resolved, functionally grounded signature for liver tumor classification.

### HCC‐TCG predicts recurrence or treatment responses

**Figure 4:**
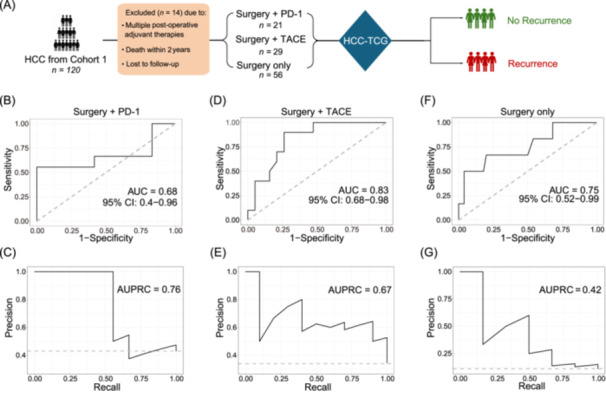
HCC‐TCG as a biomarker to predict recurrence risk in resectable HCC patients. (A) Study design on the capacity of HCC‐TCG to predict recurrence risk in surgically resectable HCC patients receiving PD‐1, TACE only, or no therapy following surgery. (B–G) Random Forest predicting model trained on the 142 HCC‐TCG genomes to distinguish recurrence and no‐recurrence among patients in the Surgery + PD‐1 group (B, C), Surgery + TACE group (D, E), and Surgery Only group (F, G). The performance of the model was assessed by AUROC (B, D, and F) and AUPRC (C, E, and G). Surgery + PD‐1 group: no recurrence *n* = 12, recurrence *n* = 9; Surgery + TACE : no recurrence *n* = 19, recurrence *n* = 10; Surgery Only group: no recurrence *n* = 50, recurrence *n* = 6.

Based on postoperative adjuvant therapy, which is additional treatment given after surgery to inhibit cancer recurrence, 106 out of the 120 patients in MT groups were stratified into three subgroups: PD‐1 immunotherapy (*n* = 21, referred as Surgery + PD‐1 group), TACE only (*n* = 29, referred as Surgery + TACE group) and no treatment (*n* = 56, referred as Surgery Only group). To investigate the potential of HCC‐TCG to predict recurrence or treatment responses, we used the abundances of the 142 HCC‐TCG HQMAGs in preoperative samples as input to train Random Forest classifiers to predict therapeutic response, as indicated by 2‐year recurrence status in each subgroup (Figure 4A). There were 12 no‐recurrence patients and 9 recurrence patients in the Surgery + PD‐1 group. No differences in age and gender were observed between two groups. Cirrhosis and tumor burden were also similar between the two groups of patients (Table S4). Our Random Forest model, built on the HCC‐TCG, demonstrated a moderate capacity to predict responses to PD‐1 therapy in postsurgery HCC patients. The model's predictive ability was quantified by an AUROC of 0.68 (Accuracy = 0.62) and an AUPRC of 0.76, which represents a remarkable improvement over the baseline AUPRC of 0.43 (Figure 4B,C).

In the Surgery + TACE group, there were 19 no‐recurrence patients and 10 recurrence patients. No difference in age, sex, and pretreatment clinical parameters was observed between the two groups of patients (Table S5). The classification model based on HCC‐TCG showed good predictive performance, achieving an AUROC of 0.83 (Accuracy = 0.62) and an AUPRC of 0.67, compared to a baseline of AUPRC of 0.34 (Figure 4D,E). Fifty‐six HCC patients did not receive any postoperative adjuvant therapy during the first 2 years after surgery. Among them, 89.28% did not have recurrence within the first 2 years. Patients with recurrence and no‐recurrence showed no difference in age, sex and cirrhosis and tumor burden (Table S6). The classification model based on HCC‐TCG had a moderate capacity to predict recurrence after surgery without other therapy. It achieved an AUROC of 0.75 (Accuracy = 0.89), and an AUPRC of 0.42, compared to a baseline of AUPRC of 0.11 (Figure 4F,G). To benchmark the performance of the HCC‐TCG model against traditional taxonomic approaches, we constructed a Random Forest model using the top 10 most differentially abundant species between BT and MT (Figure S6A,B). While this model achieved a reasonable AUROC of 0.756 in distinguishing BT from MT (Figure S6C). Its performance in recurrence prediction across the three postoperative subgroups was consistently inferior to that of the HCC‐TCG model. Specifically, the taxon‐based model achieved AUROCs of 0.514 in the Surgery + PD‐1 group, 0.753 in the Surgery + TACE group, and 0.770 in the Surgery Only group (Figure S6D–F), all lower than or comparable to the TCG‐based classifier. This highlights the superior generalizability and prognostic relevance of the ecologically structured HCC‐TCG framework.

### HCC‐associated clinical parameters enhance treatment prediction

**Figure 5:**
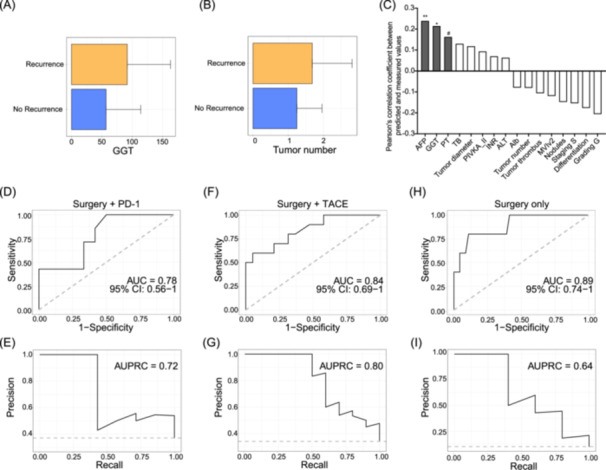
HCC associated clinical parameters enhance treatment prediction. (A, B) Bar plot showed the values of (A) gamma‐glutamyl transferase (GGT) and (B) Tumor number between the HCC patients with and without recurrence within the first 2 years after surgery. Mann–Whitney test (two‐sided) was used to analyze the difference. Recurrence *n* = 25, No recurrence *n* = 81. (C) Prediction of bio‐clinical parameters with the 142 genomes in the HCC‐TCG. Random Forest regression model was used with leave‐out‐one cross‐validation. The bar plot shows Pearson's correlation coefficient between the predicted clinical parameters and measured values. (D–I) Random Forest predicting model trained on HCC‐TCG together with GGT, alpha‐fetoprotein (AFP), prothrombin time (PT), and tumor number to distinguish nonresponder and responder among patients in the Surgery + PD‐1 group (D, E), Surgery + TACE group (F, G), and Surgery Only group (H, I). The performance of the model was assessed by AUROC (D, F, H) and AUPRC (E, G and I), the gray dashed line indicates the baseline AUPRC corresponding to the recurrence prevalence). Surgery + PD‐1 group: no recurrence *n* = 12, recurrence *n* = 9; Surgery + TACE : no recurrence *n* = 19, recurrence *n* = 10; Surgery Only group: no recurrence *n* = 50, recurrence *n* = 6. MVIv2: microvascular invasion (MVI) stage 2; tumor staging (S), based on the TNM staging system (American Joint Committee on Cancer); tumor grade (G): based on the Edmondson‐Steiner grading system. ^#^ *p* < 0.1, *p < 0.05, **p < 0.01, ***p < 0.001.

To evaluate the prognostic utility of conventional clinical features in different adjuvant treatment contexts, we constructed ridge‐penalized logistic regression models within each subgroup using a panel of established risk factors (logAFP, logDCP, ALT, PVTT, cirrhosis, gender, age, HBV status, tumor size, number, and BCLC stage). These models achieved limited predictive performance: AUROC = 0.565 in the Surgery + PD‐1 group, AUROC = 0.568 in the Surgery + TACE group, and AUROC = 0.55 in the Surgery Only group (Figure S7A‐C). These results suggest that conventional clinical variables alone are insufficient to robustly predict recurrence across treatment subgroups, underscoring the added value of the HCC‐TCG ecological signature in improving risk stratification. To evaluate if clinical parameters can enhance the response prediction models we built with the 142 genomes in HCC‐TCG, we found that among 16 clinical parameters measured before surgery, only gamma‐glutamyl transferase (GGT) and tumor number were significantly lower in the no recurrence than the recurrence group when combining all the sub‐groups (Figure 5A,B). This suggests that these two parameters were associated with recurrence risk.

To further identify the link between clinical parameters and the HCC‐TCG genomes, we conducted prediction‐based association analysis by predicting values of each of these 16 clinical parameters with the Random Forest regression models trained on HCC‐TCG genome data. We found that HCC‐TCG was associated with GGT, alpha‐fetoprotein (AFP), and prothrombin time (PT), three classical recurrence‐associated biomarkers. These associations were evident by the Random Forest regression models, which showed significantly positive correlations between the measured biomarker values and the values predicted by the models (Figure 5C). These findings suggest that Guild‐based microbial signatures reflect tumor‐related host biochemical states, indicating that HCC‐TCG can serve as a non‐invasive marker and microbial proxy for host physiology linked to recurrence risk. This raises the possibility that combining TCG microbiome features with associated clinical indices could improve prognostic modeling.

To this end, we further integrated the 3 HCC‐TCG associated clinical parameters, that is, GGT, AFP, PT, and tumor number with the HQMAGs in HCC‐TCG to evaluate whether such combination could enhance recurrence prediction. In the Surgery + PD‐1 group, the AUROC value of the Random Forest predictive model increased from 0.68 (Accuracy = 0.58) to 0.78 while AUPRC decreased slightly from 0.76 to 0.72 (Figure 5D,E). In the Surgery + TACE group, AUROC value increased slightly from 0.83 (Accuracy = 0.59) to 0.84, and AUPRC increased from 0.67 to 0.8 (Figure 5F,G). In the Surgery Only group, the AUROC increased from 0.75 (Accuracy = 0.90) to 0.89, accompanied by an increase in AUPRC from 0.42 to 0.64 (Figure 5H,I). These improvements, although modest, support the complementary nature of microbiome and clinical data in refining predictive precision.

Taken together, these results highlight the utility of a combined microbiome‐clinical framework. HCC‐TCG, when complemented by relevant clinical biomarkers, may offer an enhanced, multi‐dimensional tool for stratifying HCC patients by recurrence risk and guiding postoperative surveillance strategies.

### External validation of HCC–TCG for anti‐PD‐1 response in advanced stage HCC cohorts

**Figure 6:**
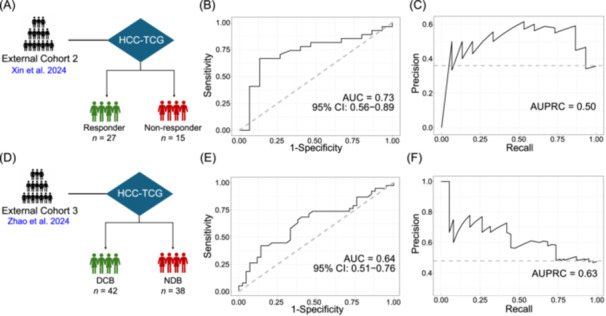
External validation of the HCC–TCG‐based response prediction model in two independent anti‐PD‐1‐treated HCC cohorts. (A) Predictive modeling workflow using HCC‐TCG in cohort from Xin et al. (2024). (B) ROC curve showing classification of responders (*n* = 27) and nonresponders (*n* = 15) (C) Precision–recall (PR) curve for the same cohort. (D) Predictive modeling workflow using HCC‐TCG in cohort from Zhao et al., 2024. (E) ROC curve for classification of durable clinical benefit (DCB, *n*  =  42) versus nondurable benefit (NDB, *n* = 38). (F) PR curve for the same cohort.

To further assess the generalizability of the HCC‐TCG in predicting treatment response, we estimated the abundance of the 142 HQMAGs of HCC‐TCG in two independent nonsurgical cohorts of advanced HCC patients receiving anti‐PD‐1‐based combination therapy. In the first cohort of 42 patients, treatment response was assessed by mRECIST, identifying 27 responders (CR/PR) and 15 nonresponders (SD/PD) [21]. All 142 HQMAGs of HCC‐TCG had an estimated abundance within this dataset, with an average prevalence of 89.70%. These genomes accounted for 24.78% of the total abundance in pretreatment fecal samples. The Random Forest classifier built on HCC‐TCG achieved an AUROC of 0.73 (Accuracy = 0.64) and an AUPRC of 0.50 (Figure 6A–C).

In the second clinical trial cohort of 80 patients with advanced HCC treated with PD‐1/PD‐L1 inhibitors with or without targeted agents, patients were stratified as having durable (DCB = 42) or non‐durable (NDB = 38) clinical benefit [22]. All 142 HQMAGs of HCC‐TCG had an estimated abundance within this dataset, with an average prevalence of 84.20%. These genomes accounted for 27.76% of mapped reads in the pretreatment samples. The Random Forest classifier built on HCC‐TCG had an AUROC of 0.64 and an AUPRC of 0.63 to predict DCB versus NDB. Together, these findings demonstrate the reproducibility and cross‐cohort utility of the HCC‐TCG as a microbiome‐based, non‐invasive predictor of therapeutic response across multiple independent populations and treatment regimens (Figure 6D–F).

To assess whether the observed discriminatory signal depends on the classifier choice, we benchmarked Random Forest against SVM and XGBoost using the same fixed 142‐HQMAG feature set across the major tasks. Performance was broadly comparable and varied by dataset, indicating that the predictive signal is not driven by a single modeling approach (Table S7).

## DISCUSSION

By focusing on microbial genomes that maintain stable relationships across benign and malignant states, rather than taxa that fluctuate with disease, we identified 142 genomes that remain persistently connected. Full taxonomic annotations are provided to facilitate independent biological interpretation; however, Guild structure itself is defined by stable genome–genome connectivity rather than taxonomic rank. These genomes self‐organize into TCG with sharply contrasting functional capacities—one enriched for pathways linked to acetate and butyrate production, and the other for traits associated with virulence, multidrug resistance, and stress‐adaptive metabolism. This HCC‐TCG core structure provides a stable, genome‐resolved ecological framework that predicts malignant state, early recurrence after surgery, and immunotherapy response across multiple independent cohorts.

Our findings extend the ecological principle established in our recent finding: stable relationships, not fluctuating taxa, define the core structure of the human gut microbiome [23]. In the earlier work, stable genome–genome correlations across diet interventions and 15 disease stages across 38 datasets revealed a universal TCG backbone associated with health [18]. Building on our previous work, we suspected malignant transformation itself serves here as a new type of perturbation—strong enough to expose the stable core of the gut ecosystem in the context of hepatic tumors. The fact that the same stability principle holds in an oncologic setting is notable. The objective of this study was not to define a cancer‐specific microbiome signature relative to healthy individuals, but to evaluate whether a stable genome‐level ecological structure persists and remains discriminatory within clinically relevant liver tumor populations. Here, we showed that the stable Guild architecture is not limited to metabolic diseases or broad health–disease contrasts, while it is a general organizational rule of the microbiome as a complex adaptive system (CAS) [23]. The emergence of the HCC‐TCG from a completely different perturbation—BT versus MTs instead of diet or disease versus healthy—suggests that Guild competition is a universal property of how the gut ecosystem organizes itself under stress. Because the TCG framework identifies stably connected genomes based on replicated connectivity patterns rather than differential abundance alone, biological heterogeneity within the BT cohort constitutes a stringent test of structural persistence rather than a confounding factor. Malignancy does not create a new microbiome structure, it reveals one that was already present, a stable ecological scaffold that changes in dominance rather than identity. This idea is consistent with the CAS view of biological systems: essential components maintain their relationships even as the environment shifts [18]. Future studies incorporating healthy and chronic liver disease cohorts will be valuable for dissecting mechanistic specificity; however, such comparisons are not required for evaluating structural transferability within a clinically relevant diagnostic context. The HCC‐TCG therefore represents an organ‐specific realization of the same ecological rule defined in the global TCG model, now contextualized within the physiology of the gut–liver axis.

Most previous microbiome studies in HCC relied on differential abundance of species or genera to identify “HCC‐associated taxa.” These studies have produced valuable observations—changes in *Enterobacteriaceae*, *Proteobacteria*, certain *Streptococcus* species, bile acid modifying microbes, and more—but the results have rarely converged across cohorts, etiologies, and sequencing platforms [24, 25, 26]. This inconsistency reflects the limitations of taxon‐based approaches: reliance on database classification, loss of strain‐level functional diversity, and a failure to account for microbial interactions [11, 15]. Our study addresses these limitations by shifting the analytic unit from taxa to genomes and from abundance shifts to stability of relationships.

To our knowledge, no previous study has reconstructed prevalent HQMAGs de novo, systematically screened them for stable correlations shared across benign and malignant hepatic tumor states, and then derived a structural Guild backbone from these persistent interactions. This methodological shift yields a reproducible ecological module, HCC‐TCG, which generalizes to multiple classification scenarios, including BT versus HCC, HCC versus CRLM, and HCC versus iCCA vs healthy controls. However, given the limited sample size in the CRLM cohort (*n* = 7), analyses involving this group were defined as exploratory, and results should be interpreted with caution. In our cohorts, HCC cases mainly included HBV‐associated (HBsAg‐positive) and non‐HBV (HBsAg‐negative) patients. Etiology‐stratified validation (e.g., HBV vs. non‐HBV, metabolic‐associated HCC) is an important future direction but is underpowered in the current cohort. The observation that both HCC and iCCA exhibit a shift toward C1B suggests a shared malignant‐associated ecological pattern rather than disease‐specific exclusivity of a single Guild. The HCC‐TCG model identifies tumor‐specific microbial dysregulation in the gut‐liver axis, which is influenced by liver tumorigenesis rather than systemic cancer‐related effects. Both CRLM and iCCA influence the gut microbiome through tumor‐driven physiological changes, such as immune signaling, metabolic stress, and bile acid alterations, creating distinct microbial selection pressures that differentiate these tumor types in the model.

Previous recurrence‐prediction studies in HCC similarly relied on differential species enrichment or diversity indices, none revealed a stable ecological architecture that spans multiple tumor types, stages, and treatment contexts [27]. By focusing on stability correlations rather than simple taxa abundance, we unify a single ecological framework that explains not only who has cancer, but who will recur or resistant to therapy. While we observed declines in predictive performance in certain therapeutic subgroups, these likely reflect the biological and clinical heterogeneity inherent to treatment response rather than a collapse of the ecological feature space. Although predictive performance decreased in certain therapeutic subgroups, the persistence of above‐chance classification using an unchanged genome set indicates retention of structural ecological signal under heterogeneous clinical conditions. In real‐world clinical settings, even moderate discrimination (AUC ≈ 0.6) can offer incremental value when integrated with established clinical factors, particularly for biologically heterogeneous endpoints, such as ICI response or postoperative recurrence risk [18, 28]. We acknowledge that larger cohorts will be essential to further confirm and enhance the reproducibility of the predictive models across these subgroups.

The functional polarity between the two Guilds, C1A and C1B, provides a coherent ecological interpretation for how the gut microbiome affects both hepatocarcinogenesis and treatment outcomes. The Guild C1A enriched for acetate and butyrate pathways, functions known to support mucosal integrity and restrain excessive inflammation, thereby limiting microbial product translocation into portal circulation and helping to prevent the establishment of a chronically pro‐inflammatory microenvironment [15, 29, 30]. The opposing Guild, C1B, is enriched in multidrug resistance determinants, virulence factors, and metabolic pathways that confer redox flexibility and enable aromatic compound processing—features characteristic of microbes adapted to stressed or chronically inflamed environments [31, 32]. These functions have been linked to host redox regulation, immune modulation, and epigenetic remodeling in liver cancer contexts, suggesting that the C1B‐enriched microbiota may contribute to a tumor‐promoting microenvironment via the gut–liver axis [33].

HCC‐TCG can be viewed as a stable ecological reorganization of the gut microbiome in response to tumor‐associated host perturbations along the gut–liver axis [34]. A shift toward C1B may reflect a tumor‐conditioned environment shaped by portal inflammation, immune dysregulation, metabolic stress, and altered SCFAs, which collectively increase ecological pressure for stress‐tolerant communities, with C1A might support immune responsiveness and C1B might promote immune resistance [35, 36]. This ecological contrast might explain how HCC‐TCG predicts both recurrence and therapeutic response, that is before treatment begins, the Guild balance likely preconditions the hepatic immune landscape. However, adjuvant treatment assignment was not randomized in these cohorts, these findings should be interpreted as associative risk stratification rather than causal evidence of treatment benefit.

A key clinically practical strength of this work is that HCC‐TCG features were derived from preoperative stool samples. The ability to predict early recurrence suggests that the HCC‐TCG structure captures underlying host–microbe interactions that persist beyond surgical resection. In other words, patients with a detrimental Guild bias may be candidates for intensified surveillance, earlier initiation of adjuvant therapy, or combination immunotherapy strategies, thereby creating new opportunities for risk stratification and tailored clinical management. Integration with clinical parameters (e.g., AFP, GGT, PT, and tumor number) amplifies this predictive accuracy, capturing host‐tumor‐microbiome interaction. These findings highlight the potential of a hybrid modeling framework—integrating ecological and physiological dimensions—to improve recurrence risk stratification in early‐stage HCC.

A long‐term clinical translational issue is whether this Guild balance can be manipulated in a way that benefits patients. Our earlier clinical trials have found that dietary fiber formulations and other nutritional strategies can selectively promote SCFA‐producing Guilds in metabolic diseases [18, 37]. However, bile acid–related pathways did not show consistent, Guild‐specific differences between C1A and C1B (data did not show). Whether similar interventions can shift the HCC‐TCG balance toward a more favorable state, even before surgery or during immunotherapy, remains an important and valuable question. Guild‐targeted nutrition and even Guild transplantation strategies may represent new ways for adjuvant or neoadjuvant therapy [18, 37]. Prospective trials incorporating Guild analysis at multiple timepoints would allow us to observe ecological transitions during treatment and determine whether Guild shifts precede clinical outcomes. These studies could help establish HCC‐TCG as both a predictive biomarker and a modifiable therapeutic target, moving microbiome‐based strategies from association to intervention [38].

Our study is based on fecal metagenomes, which reflect distal gut ecology but may not capture microbial communities in the small intestine or bile ducts [39]. Functional predictions are based on genome content rather than direct metabolomic or immunologic measurements. Longitudinal studies integrating microbiome, metabolome, bile acids, and immune phenotyping will be essential to validate the ecological pathways proposed here [14, 40]. Mechanistic experiments—particularly those testing whether shifts toward the more favorable Guild improve treatment outcomes—will strengthen causal interpretation [37, 41, 42]. We acknowledge that alternative machine‐learning algorithms may yield incremental gains as larger and more diverse datasets become available. Nevertheless, the central aim of this work is to evaluate the reproducibility and interpretability of a stable Guild‐based microbiome representation, rather than to pursue maximal performance through exhaustive algorithmic tuning. The consistent HCC‐TCG–based performance across tumor classification, recurrence prediction, and treatment‐response settings supports this conclusion.

## CONCLUSION

In summary, the HCC‐TCG framework shows that a stable, antagonistic Guild structure of the gut microbiome underlies malignant transformation, recurrence risk, and immunotherapy response along the gut–liver axis. This study extends the TCG paradigm into hepatic oncology and provides a structurally grounded, functionally interpretable, and clinically actionable ecological signature for HCC. By revealing a stable microbial architecture that spans benign and malignant states, primary and metastatic tumors, and surgical and immunotherapy settings, this work lays the foundation for a new class of Guild‐based diagnostics and interventions in liver cancer.

## METHODS

### Local clinical cohorts

This study included two local clinical cohorts. Cohort 1 consisted of benign hepatic tumors cases (BT, *n* = 76) and surgically resectable HCC cases (HCC, *n* = 120), who underwent surgical resection at Zhongshan Hospital, Fudan University, between November 2019 and April 2021.

This cohort was used to develop a microbiome‐based classifier to distinguish malignant liver tumors from benign liver lesions and predict recurrence following adjuvant therapy in a clinically relevant preoperative scenario. Surgically treated benign liver lesions were used as controls to ensure consistent clinical workup and sample collection, minimizing biases from differences in healthcare access and examination intensity between surgical patients and community healthy volunteers. Cohort 2 comprised HCC cases (*n* = 33) and colorectal liver metastases cases (CRLM, *n* = 7) who were diagnosed and treated at Ningbo No. 2 Hospital between January 2023 and December 2024.

HCC diagnosis was confirmed histopathologically from resected specimens. Treatment decisions followed international guidelines through multidisciplinary team (MDT) discussions, with resection performed for early‐stage HCC in patients with preserved liver function (Child–Pugh class A or B) and sufficient future liver remnant [2]. All included patients had postoperative confirmation of HCC and underwent R0 resection, defined as no residual tumor with negative microscopic margins ≥1 cm. Patients were excluded if they had extrahepatic metastasis, received any prior anti‐cancer therapy, or had other hepatic malignancies or a history of other cancers.

Adjuvant Therapies: Postoperative adjuvant treatments were administered based on MDT risk assessment [43]. (1) TACE: Per clinical guidelines, TACE was administered 4 weeks after resection in high‐risk patients. Procedures were performed via the Seldinger technique, with the choice of chemotherapeutic agents and their dosages tailored according to the individual patient's condition by the attending physician [44]. (2) Anti‐PD‐1 therapy: ICI was offered as adjuvant therapy based on recurrence risk and patient preference. PD‐1 inhibitors were administered intravenously every 21 days and continued until disease progression or unacceptable toxicity [45].

BT controls comprised patients who underwent hepatic resection during the same period, either due to meeting surgical indications for benign liver tumor or because preoperative imaging identified liver space‐occupying lesions for which HCC could not be excluded. Postoperative histopathological examination confirmed benign entities, including hepatic hemangioma and hepatocellular adenoma. CRLM was diagnosed based on: (1) histopathological confirmation of adenocarcinoma consistent with colorectal origin; (2) clinical history of primary colorectal carcinoma; and (3) imaging evidence (contrast‐enhanced MRI or CT) of liver lesions with typical metastatic features.

Exclusion criteria for both cohorts included: (1) Incomplete clinical data; (2) Use of systemic antibiotics or probiotic/prebiotic products within 3 months prior to fecal sample collection; (3) Undergoing colonoscopy and/or bowel preparation within 1 month prior to sampling.

For all patients, comprehensive clinical data were collected, including demographics, liver function tests, hepatitis B/C serology, tumor markers (e.g., AFP), and tumor number assessed by preoperative imaging. The study was approved by the Institutional Review Boards of Ningbo No. 2 Hospital (PJ‐NBEY‐KY‐2024‐189‐01) and Zhongshan Hospital, Fudan University (B2019‐060R). A brief cross‐check table in the Supplementary Materials (Table S8), listing sample IDs in these two cohorts by group and end point, to further aid clarity and reproducibility.

### Clinical endpoints

Patients were followed every 1–2 months for the first 6 months post‐discharge, then every 3–6 months. The primary clinical endpoint was early recurrence, defined as intrahepatic or extrahepatic tumor relapse within 24 months from the date of surgery, confirmed by contrast‐enhanced CT or MRI. In the Surgery Only group, recurrence was defined as 2‐year recurrence. For patients who received adjuvant therapy and experienced recurrence within this time frame, they were classified as nonresponders (recurrence occurred despite treatment).

### Fecal sample collection

Fecal samples were collected from all patients after admission but before surgery, following standardized protocols, and stored at −80°C until DNA extraction.

### Metagenomic sequencing

Microbial DNA from fecal samples were extracted using QIAamp DNA Stool Mini Kit (TIANGEN Biotech) according to manufacturer's protocol. Metagenomic library preparation and sequencing were conducted by a sequencing service provider, Majorbio, using a verified NGS workflow (prepared DNA insert ~500 bp) and PE150 sequencing on a HiSeq 3000 platform. Mean sequencing depth per sample was 24.30 ± 2.58 million (mean ± SD) read pairs.

### Data quality control

KneadData (https://huttenhower.sph.harvard.edu/kneaddata/) was applied to perform quality control of the raw reads with the following parameters: decontaminate‐pairs strict, ‐run‐trim‐repetitive, ‐bypass‐trf, and ‐trimmomatic‐options = “slidingwindow:4:20 minlen:60.” Reads that could be aligned to the human genome (hg37 genome) were identified and removed in KneadData. On average, 22.61 ± 2.61 million (mean ± SD) high‐quality read pairs remained for further analysis.

### De novo assembly, abundance calculation, and taxonomic assignment of genomes

High‐quality reads were assembled into contigs in each sample using MEGAHIT (‐min‐contig‐len 500 and ‐preset meta‐large) [46], binned with MetaBAT2 [47] and MaxBin2 [48], and refined using the bin_refinement module from MetaWRAP [49] to combine and improve the results generated by the two binners. The quality of the bins was assessed using CheckM2 [50]. Bins with completeness >95% and contamination <5% were retained as high‐quality draft genomes, which were further dereplicated using dRep [51]. DiTASiC [52], which applies kallisto for pseudo‐alignment [53] and a generalized linear model for resolving shared reads among genomes, was used to calculate the abundance of the genomes in each sample, and estimated counts with *p* > 0.05 were removed. GTDB‐Tk (database v214.1) [54] was used to conduct taxonomic assignment of the obtained genomes.

### Gut microbiome network construction and analysis

Prevalent HQMAGs in >75% samples in both BT and MT groups were used to build co‐abundance networks using FastSpar (1000 permutations, BH adjusted *p* < 0.05 retained). Networks visualized in Cytoscape v3.8.1 with edge‐weighted spring‐embedded layout (correlation coefficients as weights).

### Identification of the TCGs

Robust, stable edges were defined as the unchanged positive/negative correlations between the same two genomes across BT and MT groups. The same clustering analysis, performed in our previous research, was conducted on the stable network [18].

### External cohorts and data

To validate the robustness of the classification and prediction capacity of HCC–TCG genomes across diverse populations and clinical contexts, we analyzed three independent fecal metagenomic datasets. Two datasets were obtained from the European Nucleotide Archive (ENA): a Thai cohort (PRJNA932948) including patients with HCC (*n* = 25), intrahepatic cholangiocarcinoma (iCCA, *n* = 19), and healthy controls (CTR, *n* = 76) [20]; and a Chinese cohort (PRJNA1142390) comprising 45 HCC patients treated with anti‐PD‐1‐based combination therapy, patients were stratified 27 responders (CR/PR) and 15 nonresponders (SD/PD) [21]. In addition, we included a previously published cohort [22] involving 80 patients with advanced surgically unresectable HCC from multicenter clinical trials, treated with PD‐1/PD‐L1 inhibitors with or without molecular targeted agents. Patients were stratified into durable clinical benefit (DCB, *n* = 42) and nondurable benefit (NDB, *n* = 38) groups based on treatment response [22]. Quality control of the raw metagenomic data was conducted by KneadData. DiTASiC was used to recruit reads and estimate the abundance of the 142 HQMAGs in the HCC‐TCG in each sample, and estimated counts with *p* > 0.05 were removed. Random Forest models of each dataset were trained based on the estimated abundance of the 142 HQMAGs.

For external cohorts, we did not re‐assemble MAGs or re‐infer co‐abundance networks. Instead, the 142 HCC‐TCG genomes identified in the discovery cohorts were treated as a fixed genome‐resolved reference signature. For all external cohorts, the original 142 HQMAGs were retained without feature re‐selection. Genome abundances were quantified by read recruitment using the same pipeline as in the discovery cohort. No feature re‐selection or re‐derivation was performed in validation cohorts. Briefly, quality‐controlled reads from each external dataset were mapped to the 142 HQMAGs (genome‐level read recruitment) to estimate genome relative abundance and prevalence (defined as the proportion of samples with non‐zero mapped abundance above the detection threshold). The Guild assignment (C1A vs. C1B) was kept unchanged from the discovery stage, and C1A/C1B prevalence in each cohort was summarized as the proportion of samples in which at least one genome from the corresponding Guild was detected (and/or by the distribution of Guild‐level summed abundance). This strategy directly tests whether the same stability‐defined genomes and Guild structure are detectable and quantitatively represented across independent cohorts.

### Gut microbiome functional analysis

HQMAGs were functionally annotated with Prokka [55]; KEGG Orthology (KO) were assigned via KOfam/KofamKOALA [56]; ARGs and VFs were identified via ResFinder [57] and MetaVF [58] respectively; Genes encoding *fthfs*, *scpC*, *pct*, *4hbt*, *atoa*, *atod*, *buk* and *but* were identified as described previously [37].

### Statistics

Statistical analysis was performed in the R environment (R version 4.2.1). Continuous variables were summarized as mean ± SD for approximately normally distributed data and as median (IQR) otherwise; categorical variables were summarized as counts and percentages. Between‐group comparisons were performed using Student's *t*‐test for parametric data and two‐sided Mann–Whitney *U* test for nonparametric data. Categorical variables were compared using Pearson's chi‐square test or Fisher's exact test, as appropriate. PERMANOVA test (9999 permutations) was used to compare overall microbiota structure between the groups. Random forest classification and regression models were built on the 142 HCC‐TCG genomes with leave‐one‐out cross‐validation by using R caret and Random Forest packages. We benchmarked Random Forest against SVM and XGBoost using the same fixed 142‐HQMAG feature set across all tasks. For Guild‐level functional gene comparisons, two‐sided Mann–Whitney *U* tests were used for continuous measures and Fisher's exact test for categorical measures, as applicable. These 142 genomes formed the basis of the HCC‐TCG structure and remained unchanged across all subsequent analyses. To evaluate the predictive value of clinical variables on recurrence outcomes, models were constructed separately within each postoperative treatment subgroup (e.g., PD‐1, TACE, surgery alone). For multivariate modeling involving categorical predictors and limited sample sizes, ridge‐penalized logistic regression (L2‐regularized) was employed using R glmnet to improve model stability and reduce overfitting risk [59]. AUROC was used to evaluate the capacity of features to discriminate between groups using the R package pROC [60]. AUROC considers the trade‐offs between sensitivity and specificity and compares the performance of classifiers with a baseline value of 0.5 for a random classifier. AUPRC, which considers the trade‐offs between precision and recalls with a baseline that equals the proportion of positive cases in all samples, was used as a complementary assessment, particularly for highly imbalanced data sets via PRROC package [61]. For comparisons between two independent groups (e.g., MT vs. BT/recurrence vs. no‐recurrence), Student's *t*‐test was applied to parametric data and the two‐sided Mann–Whitney U test to nonparametric data. Continuous variables were summarized as mean ± standard deviation (SD) for normally distributed data and as median with interquartile range (IQR) for nonnormally distributed data. Categorical variables were expressed as counts and percentages.

## AUTHOR CONTRIBUTIONS

**Yang Liu**: Conceptualization; methodology; data curation; writing—original draft. **Zefan Zhang**: data curation; resources. **Guojun Wu**: Methodology; software; validation; formal analysis. **Bowen Li**: Data curation; resources. **Linghua Wang**: Writing—review and editing. **Jincheng Wang**: Writing—review and editing. **Zixian Wei**: Data curation; resources. **Zhiyue Wang**: Formal analysis; visualization. **Jinhua Yang**: Visualization. **Kunyu Zhang**: Visualization. **Tianqi Zhang**: Visualization. **Xin Tao**: Investigation. **Tao Chen**: Investigation. **Jia Fan**: Funding acquisition; supervision. **Jian Zhou**: Supervision; funding acquisition. **Xinrong Yang**: Funding acquisition; supervision; resources. **Liping Zhao**: Conceptualization; methodology; writing—review and editing. **Yunwei Wei**: Conceptualization; methodology; project administration; funding acquisition; writing—review and editing. All authors have read the final manuscript and approved it for publication.

## CONFLICT OF INTEREST STATEMENT

Liping Zhao is co‐founder of Notitia Biotechnologies Company. The other authors declare no conflict of interest.

## ETHICS STATEMENT

The study was approved by the Institutional Review Boards of Ningbo No. 2 Hospital (PJ‐NBEY‐KY‐2024‐189‐01) and Zhongshan Hospital, Fudan University (B2019‐060R). All eligible patients provided written informed consent and were enrolled prior to surgery.

## Supporting information


**Figure S1.** Comparison of gut microbiome alpha diversity between benign and malignant liver tumor patients.
**Figure S2.** Stable correlations of microbial genomes across benign and malignant tumor groups.
**Figure S3.** Stable ecological correlations organize the core microbiome into two competing guilds.
**Figure S4.** Total abundance of C1A and C1B were similar between BT and MT groups.
**Figure S5.** Total abundance of C1A and C1B were similar between CRLM and HCC groups.
**Figure S6.** Performance of a taxon‐based classifier using top 10 differentially abundant species.
**Figure S7.** Prognostic Value of clinical variables in predicting HCC recurrence after surgery.

**Table S1.** Demographic and clinical characteristics of benign tumor (BT) vs malignant tumor (MT) groups.
**Table S2.** Genome IDs and guild membership of the 142 HQMAGs in the HCC‐TCG.
**Table S3.** Demographic and clinical characteristics of HCC vs CRLM groups.
**Table S4.** Demographic and clinical characteristics of Surgery + PD‐1 subgroup.
**Table S5.** Demographic and clinical characteristics of Surgery + TACE subgroup.
**Table S6.** Demographic and clinical characteristics of Surgery only subgroup.
**Table S7.** Benchmarking RF, SVM, and XGBoost on the fixed 142‐HQMAG HCC‐TCG feature set.
**Table S8.** Sample ID, cohort assignment, and endpoint labels.

## Data Availability

The metagenomic sequencing data generated from the local study cohorts in this work have been deposited in the NCBI Sequence Read Archive under accession number PRJNA1372073, https://www.ncbi.nlm.nih.gov/Traces/study/?acc=PRJNA1372073&o=acc_s%3Aa and PRJNA1372070, https://www.ncbi.nlm.nih.gov/Traces/study/?acc=PRJNA1372070&o=acc_s%3Aa. Clinical metadata were not publicly available due to legal and informed consent restrictions. Reasonable requests to access the data sets should be directed to the corresponding authors, Yunwei Wei and Xinrong Yang. The external validation datasets used in this study are publicly available: Thai cohort including HCC, iCCA, and healthy controls: NCBI SRA accession PRJNA932948, https://www.ncbi.nlm.nih.gov/Traces/study/?acc=PRJNA932948&o=acc_s%3Aa. Chinese cohort of HCC patients receiving anti–PD‐1 therapy: NCBI SRA accession PRJNA1142390, https://www.ncbi.nlm.nih.gov/Traces/study/?acc=PRJNA1142390&o=acc_s%3Aa. Additional immunotherapy cohorts were obtained from previously published studies. The data and scripts used in this study are hosted on GitHub at https://github.com/nightkid03/HCC-TCG. Supplementary materials (figures, tables, graphical abstract, slides, videos, Chinese translated version and update materials) may be found in the online DOI or iMeta Science (http://www.imeta.science). The data that support the findings of this study are openly available in NCBI SRA at https://www.ncbi.nlm.nih.gov/sra, reference number PRJNA1372070; PRJNA1372073.
