# Supplementary material for: Two stable gut microbiome guilds predict liver tumor class and treatment responses
Source: Imeta. 2026 Apr 2;5(2):e70123. doi: 10.1002/imt2.70123 (PMC13147943; doi:10.1002/imt2.70123)
Supplement: Supplementary file 1 — Figure S1. Comparison of gut microbiome alpha diversity between benign and malignant liver tumor patients. Figure S2. Stable correlations of microbial genomes across benign and malignant tumor groups. Figure S3. Stable ecological correlations organize the core microbiome into two competing guilds. Figure S4. Total abundance of C1A and C1B were similar between BT and MT groups. Figure S5. Total abundance of C1A and C1B were similar between CRLM and HCC groups. Figure S6. Performance of a taxon‐based classifier using top 10 differentially abundant species. Figure S7. Prognostic Value of clinical variables in predicting HCC recurrence after surgery. [file IMT2-5-e70123-s002.docx]

**Supporting information to**

**Two stable gut microbiome guilds predict liver tumor class and treatment responses**

**Running title:** A guild-based microbiome signature for HCC

Yang Liu^1,2#^, Zefan Zhang^3#^，Guojun Wu^4,5#^, Bowen Li^1,2,3#^,Linghua Wang^6^, Jincheng Wang^6^，Zixian Wei^3^，Zhiyue Wang^1,2^, Jinhua Yang^1,2^, Kunyu Zhang^1,2^, Tianqi Zhang^1,2^, Xin Tao^1,2^,Tao Chen^1,2^, Jia Fan^3^, Jian Zhou^3^, Xinrong Yang^3*^, Liping Zhao^4,5,6*^, Yunwei Wei^1,2*^

^1^Department of Hepatobiliary and Pancreatic Surgery Division, Ningbo No.2 Hospital, Wenzhou Medical University, 315000, China.

^2^Ningbo Key Laboratory of Intestinal Microecology and Human Major Diseases, Ningbo 315000, China.

^3^Department of Hepatobiliary Surgery & Transplantation, Liver Cancer Institute, Zhongshan Hospital, Fudan University; Key Laboratory of Carcinogenesis and Cancer Invasion, Ministry of Education, Shanghai 200032, China.

^4^Department of Biochemistry and Microbiology, School of Environmental and Biological Sciences and Center for Microbiome, Nutrition, and Health, New Jersey Institute for Food, Nutrition, and Health, Rutgers, The State University of New Jersey, New Brunswick, NJ 08901, USA.

^5^Rutgers-Jiaotong Joint Laboratory for Microbiome and Human Health, New Brunswick, NJ 08901, USA.

^6^State Key Laboratory of Microbial Metabolism and Ministry of Education Key Laboratory of Systems Biomedicine, School of Life Sciences and Biotechnology, Shanghai Jiao Tong University, Shanghai 200240, China

^#^These authors contributed equally: Yang Liu, Zefan Zhang, Guojun Wu and Bowen Li.

*Correspondence: [hydwyw11@hotmail.com](mailto:hydwyw11@hotmail.com) (Yunwei Wei); [liping.zhao@rutgers.edu](mailto:liping.zhao@rutgers.edu) (Liping Zhao); [yang.xinrong@zs-hospital.sh.cn](mailto:yang.xinrong@zs-hospital.sh.cn) (Xinrong Yang).

**Supplementary figures**


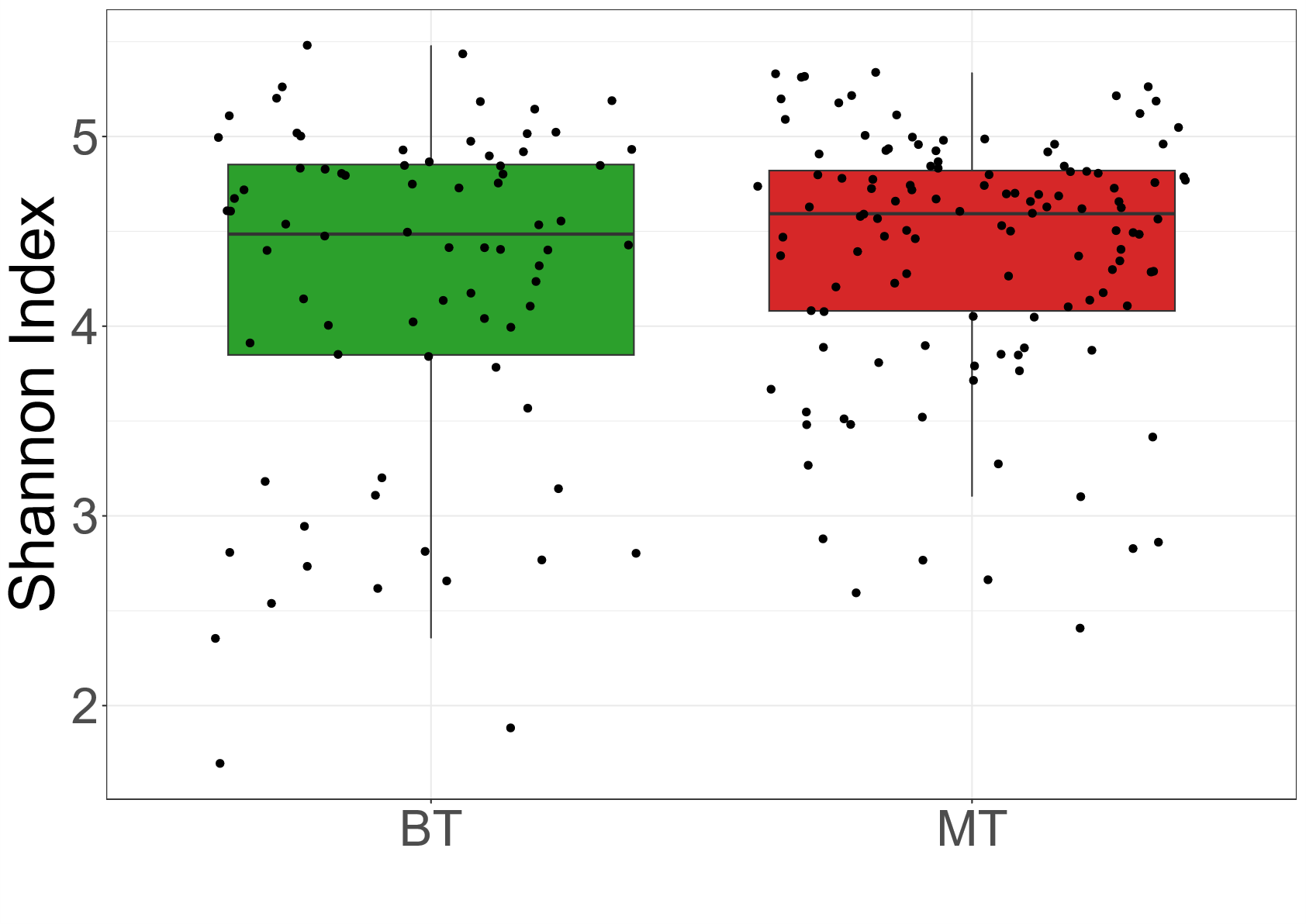


**Figure S1 Comparison of gut microbiome alpha diversity between benign and malignant liver tumor patients.** Shannon diversity index was calculated based on species-level taxonomic profiles derived from metagenomic sequencing. Each dot represents an individual sample. The box represents the interquartile range (IQR), the horizontal line indicates the median, and whiskers extend to 1.5 × IQR. Differences between groups were evaluated using the Wilcoxon rank-sum test.


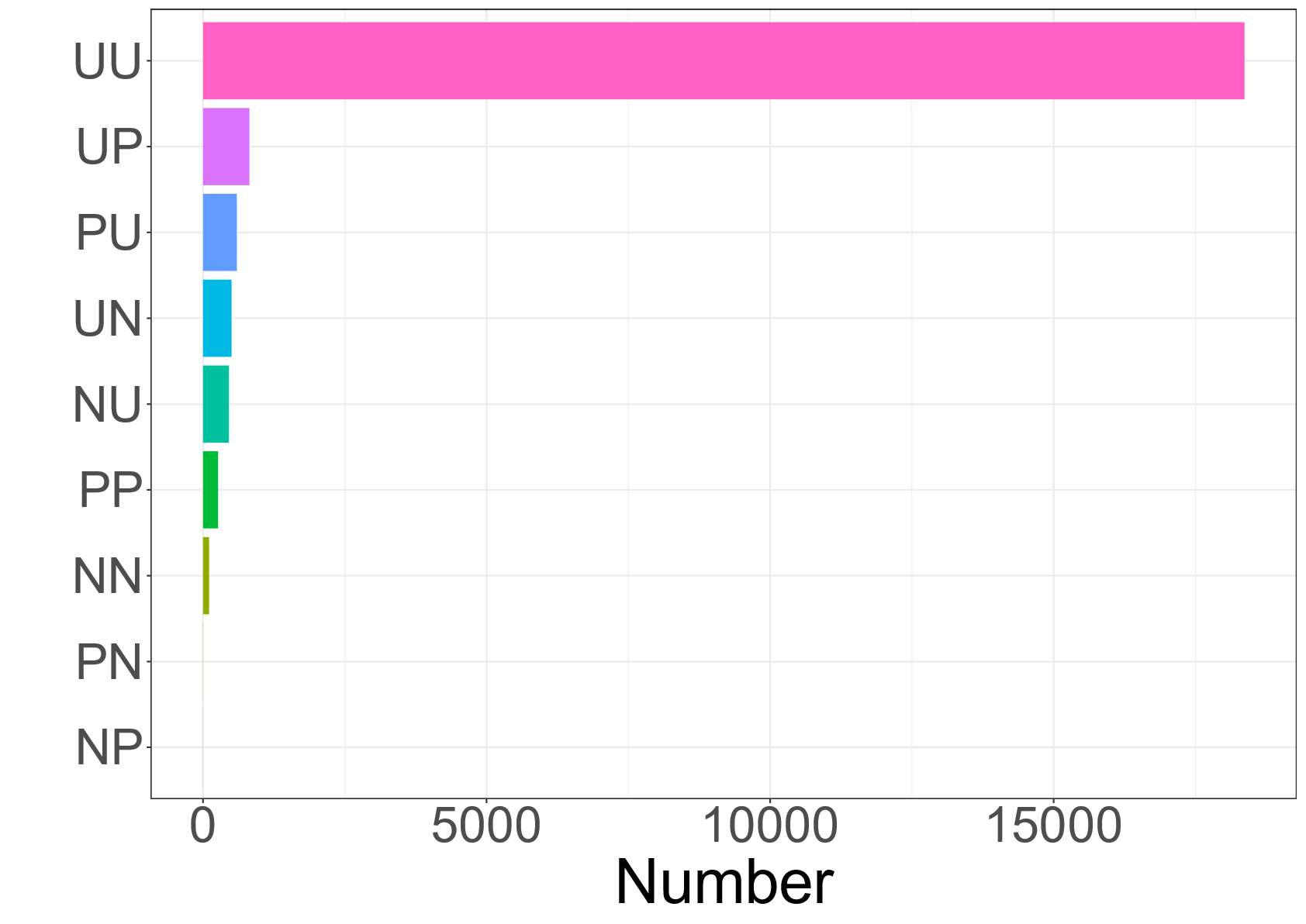


**Figure S2 Stable correlations of microbial genomes across benign and malignant tumor groups.** Bar plots showing the number of genome pairs with different correlation types across benign tumor (BT) and malignant tumor (MT) groups. U, unrelated; P, positive correlation; N, negative correlation. UP and PU indicate pairs with positive correlation in one group but unrelated in the other; UN and NU indicate pairs with negative correlation in one group but unrelated in the other; PP and NN represent consistent positive or negative correlations across both groups; PN and NP indicate opposite correlation directions between groups.


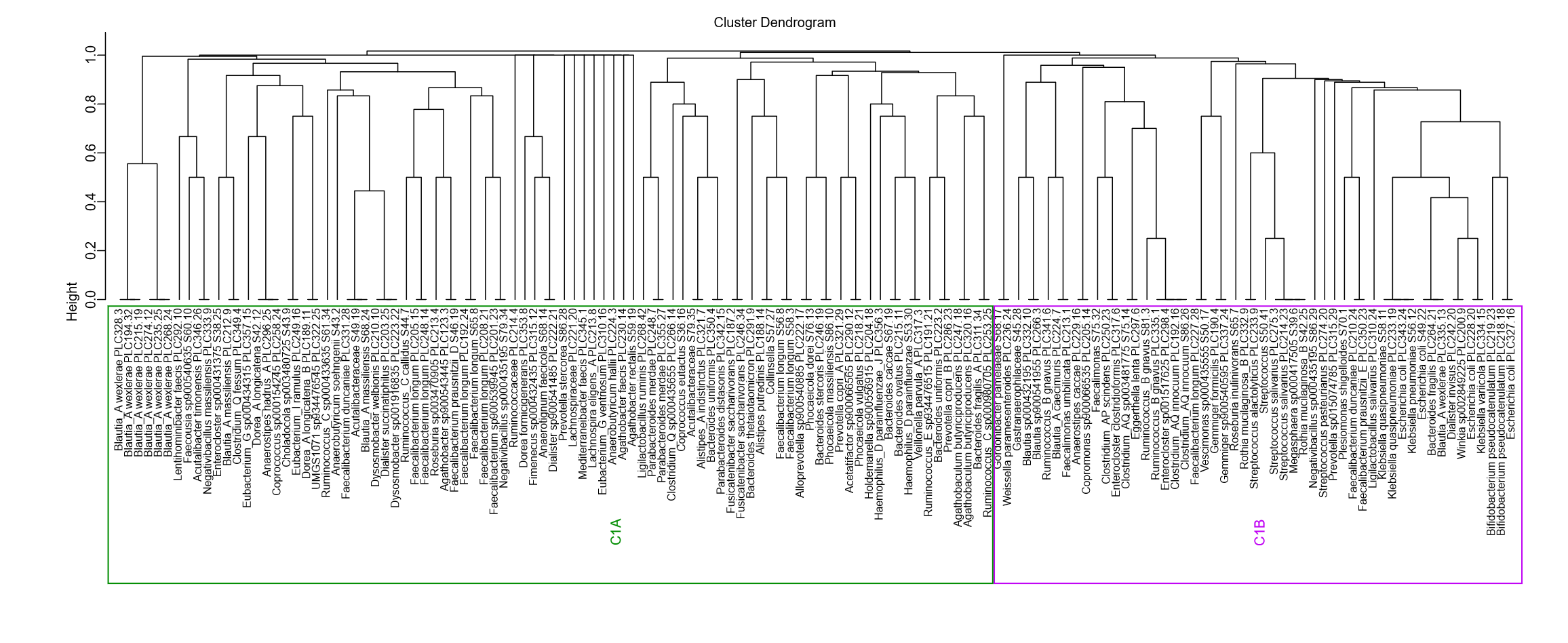


**Figure S3 Stable ecological correlations organize the core microbiome into Two Competing Guilds.**
Network analysis of 142 stably correlated high-quality metagenome-assembled genomes (HQMAGs) identified from both benign tumor (BT) and malignant tumor (MT) groups. Hierarchical clustering based on stable correlations resolved two sub-clusters, C1A and C1B, representing internally cooperative but mutually antagonistic microbial guilds.


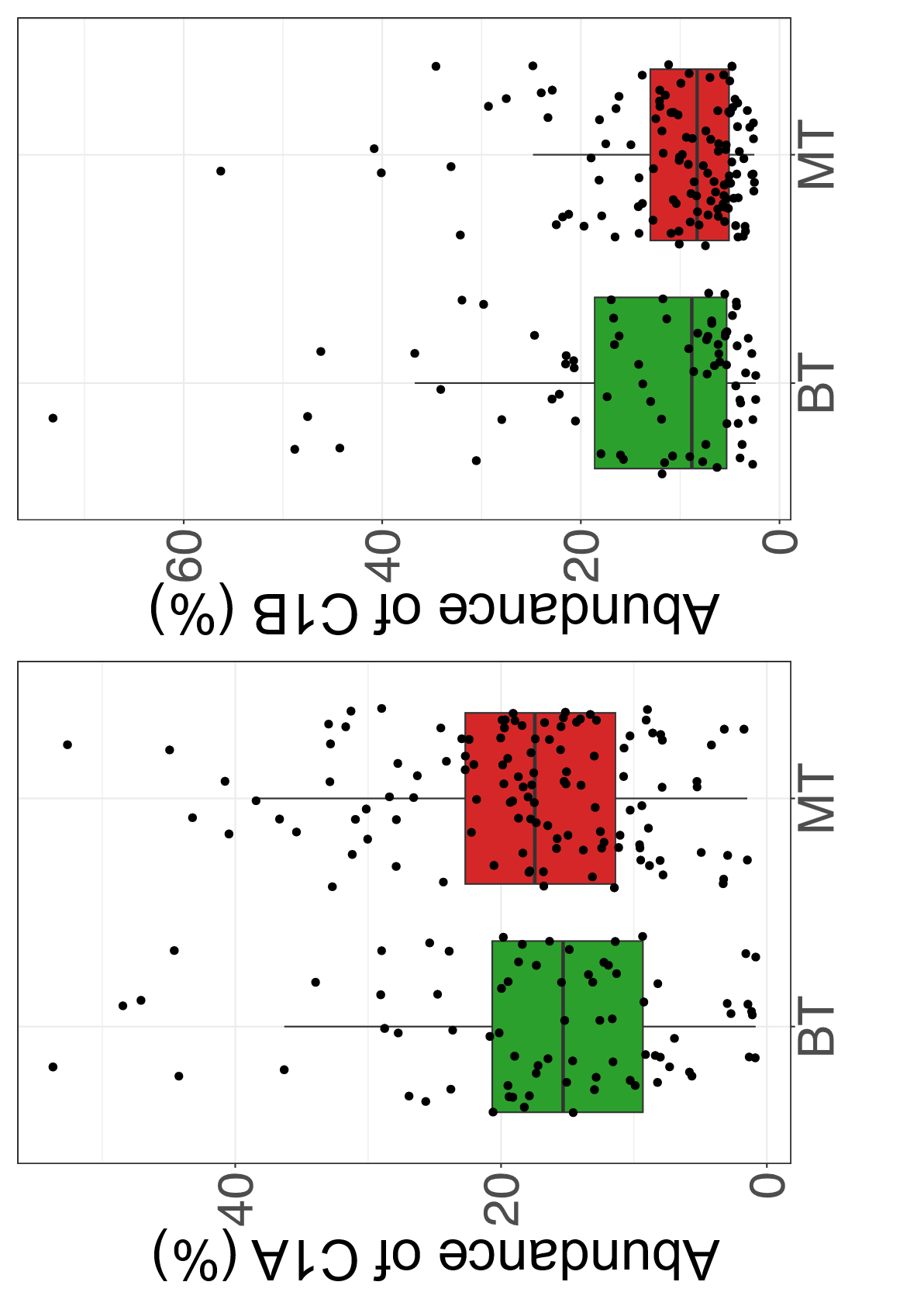


**Figure S4 Total abundance of C1A and C1B were similar between BT and MT groups.** Box plots show medians and interquartile ranges (IQRs); whiskers denote the lowest and highest values that were within 1.5× the IQR from the first and third quartiles, and each dot represents an individual sample. Mann-Whitney test (two-sided) was used to compare groups.


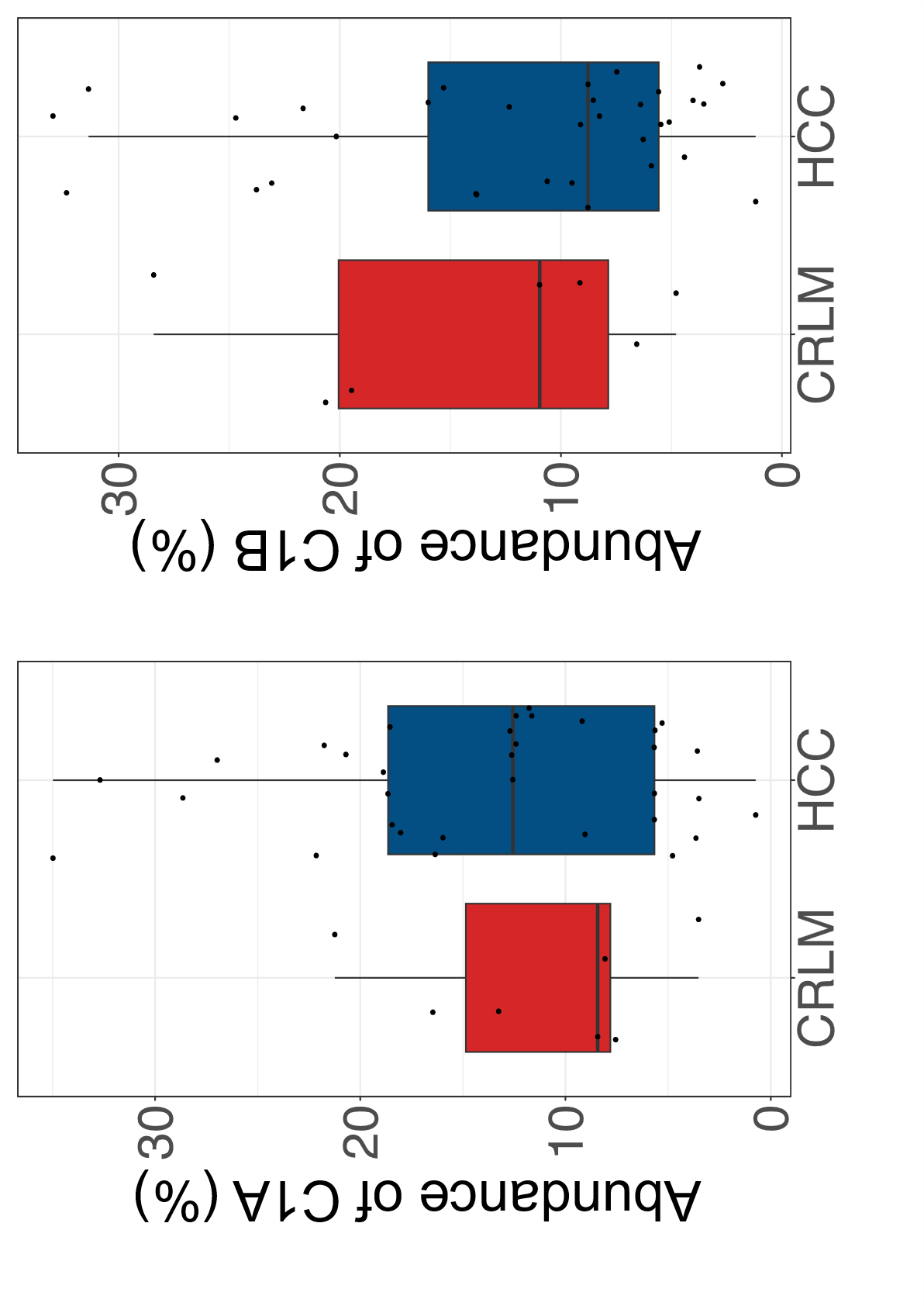


**Figure S5 Total abundance of C1A and C1B were similar between CRLM and HCC groups.** Box plots show medians and interquartile ranges (IQRs); whiskers denote the lowest and highest values that were within 1.5× the IQR from the first and third quartiles, and outliers are shown as individual points. Mann-Whitney test (two-sided) was used to compare groups.


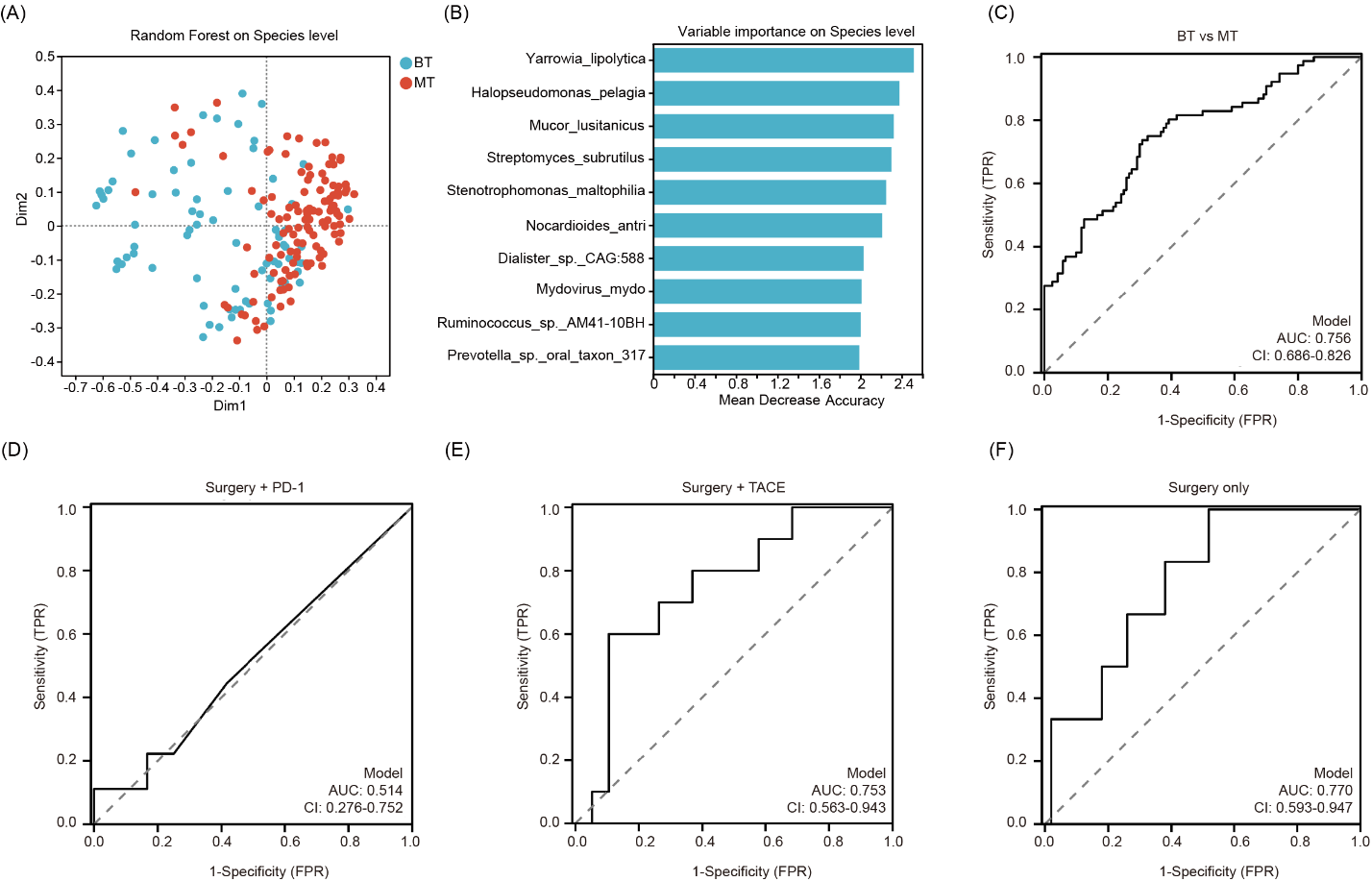


**Figure S6 Performance of a taxon-based classifier using top 10 differentially abundant species.** (A) Principal coordinates plot (PCoA) based on random forest proximity matrix showing separation between benign tumor (BT, blue) and malignant tumor (MT, red) samples using the top 10 differentially abundant species. (B) Variable importance plot ranked by mean decrease in accuracy in the random forest model, highlighting key microbial taxa contributing to the classification. (C) ROC curve for BT vs MT classification using the taxon-based model, yielding an AUROC of 0.756 (95% CI: 0.686–0.826). (D–F) ROC curves for recurrence prediction in three postoperative subgroups using the same taxon-based model: (D) Surgery + PD-1 immunotherapy subgroup (AUROC = 0.514, CI: 0.276–0.752). (E) Surgery + TACE subgroup (AUROC = 0.753, CI: 0.563–0.943), (F) Surgery-Only subgroup (AUROC = 0.770, CI: 0.593–0.947). Although the taxon-based classifier achieved moderate performance in distinguishing BT from MT, it performed less robustly in recurrence prediction compared to the HCC-TCG–based model.


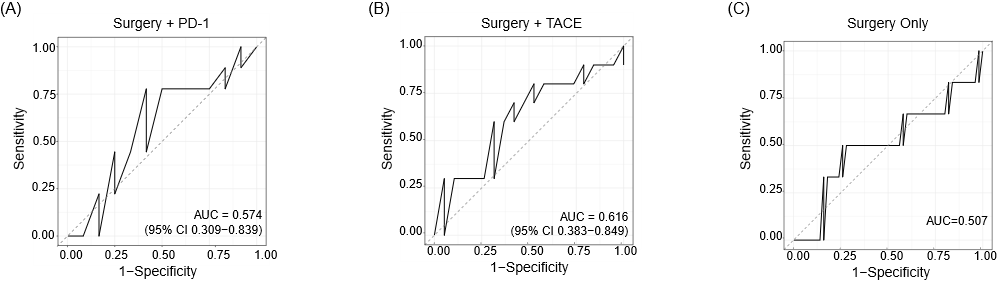


**Figure S7 Prognostic value of clinical variables in predicting HCC recurrence after surgery.** Ridge-penalized logistic regression models were built separately for each adjuvant therapy subgroup using key clinical variables (logAFP, logDCP, ALT, PVTT, cirrhosis, gender, age, HBV, tumor size, number, and BCLC stage) to predict 2-year recurrence following surgery. (A) Surgery + PD-1 group: AUROC = 0.565 (95% CI: 0.306–0.815). (B) Surgery + TACE group: AUROC = 0.568 (95% CI: 0.321–0.795). (C) Surgery Only group: AUROC = 0.550 (95% CI: 0.253–0.817).
